# Predicting the potential global distribution of an invasive alien pest *Trioza erytreae* (Del Guercio) (Hemiptera: Triozidae)

**DOI:** 10.1038/s41598-022-23213-w

**Published:** 2022-11-25

**Authors:** Owusu Fordjour Aidoo, Philipe Guilherme Corcino Souza, Ricardo Siqueira da Silva, Paulo Antonio Santana Júnior, Marcelo Coutinho Picanço, Rosina Kyerematen, Mamoudou Sétamou, Sunday Ekesi, Christian Borgemeister

**Affiliations:** 1 Department of Biological, Physical and Mathematical Sciences, School of Natural and Environmental Sciences, University of Environment and Sustainable Development, Somanya, Ghana; 2 Department of Agronomy, Universidade Federal dos Vales do Jequitinhonha e Mucuri (UFVJM), Diamantina, MG 39100-000 Brazil; 3 Department of Entomology, Universidade Federal de Viçosa, Av. P. H. Rolfs, s/n, Viçosa, MG 36570-900 Brazil; 4 Department of Animal Biology and Conservation Sciences (DABCS), University of Ghana, P.O. Box LG 67, Legon-Accra, Ghana; 5 Citrus Center, Texas A&M University-Kingsville, 312 N. International Blvd., Weslaco, TX 78599 USA; 6 Plant Health Theme, International Centre of Insect Physiology and Ecology (Icipe), P.O. Box 30772-00100, Nairobi, Kenya; 7 Centre for Development Research (ZEF), University of Bonn, Genscherallee 3, 53113 Bonn, Germany

**Keywords:** Biological techniques, Computational biology and bioinformatics, Ecology, Zoology, Climate sciences, Ecology, Environmental sciences, Energy science and technology

## Abstract

The impact of invasive alien pests on agriculture, food security, and biodiversity conservation has been worsened by climate change caused by the rising earth’s atmospheric greenhouse gases. The African citrus triozid, *Trioza erytreae* (Del Guercio; Hemiptera: Triozidae), is an invasive pest of all citrus species. It vectors the phloem-limited bacterium “*Candidatus* Liberibacter africanus”, a causal agent of citrus greening disease or African Huanglongbing (HLB). Understanding the global distribution of *T. erytreae* is critical for surveillance, monitoring, and eradication programs. Therefore, we combined geospatial and physiological data of *T. erytreae* to predict its global distribution using the CLIMEX model. The model’s prediction matches *T. erytreae* present-day distribution and shows that parts of the Mediterranean region have moderate (0 < EI < 30) to high (EI > 30) suitability for the pest. The model predicts habitat suitability in the major citrus-producing countries, such as Mexico, Brazil, China, India, and the USA. In the Special Report on Emissions Scenarios (SRES) A1B and A2 scenarios, the model predicts a reduction in habitat suitability from the current time to 2070. The findings show that global citrus production will continue to be threatened by *T. erytreae*. However, our study provides relevant information for biosecurity and risk assessment.

## Introduction

Biological invasion of agricultural ecosystems has increased due to global factors, such as weak economies, international trade, climate change, poor regulatory regimes, and environmental concerns^1–4^. These factors have compounded the impact of invasive species on sustainable agriculture, food security, and biodiversity conservation^5–7^. The African citrus triozid (ACT), *Trioza erytreae* (Del Guercio; Hemiptera: Triozidae), is an invasive alien pest of all citrus species. *Trioza erytreae* feeds on about 18 non-citrus host plants, all belonging to the family Rutaceae^8^. The pest spreads locally through natural dispersal up to about 1.5 km per week^9^, but long distance spread mainly has occurred as a result of trade in infested plants. It is listed as an A1 quarantine pest by the European and Mediterranean Plant Protection Organization, the Caribbean Plant Protection Commission and Organismo Internacional Regional de Sanidad Agropecuaria^10^. *Trioza erytreae* was first reported on citrus in 1922 in the South African regions of the Eastern Cape and Stellenbosch^11^. Over 28 countries from Europe, Middle East, and Africa, have reported *Trioza erytreae*^10^. In these areas, *T. erytreae* poses a threat to their citriculture^12^.

*Trioza erytreae* induces direct damage to hosts through its feeding activities. The symptomatic shoots show pit-like galls and secretion of honeydew, which have been associated with a reduction of yield and productivity of the affected plants^13^. Direct damage to hosts is important, but of more significant concern is its ability to vector the phloem-limited bacterium “*Candidatus* Liberibacter africanus”, implicated in incurable African Huanglongbing (HLB) or citrus greening disease, which represents a substantial setback to the citrus industry wherever it occurs^14^.

Climate is one of the most important factors influencing the occurrence, geographical distribution, population dynamics, and natural enemies of pests on a global scale^15–17^. Warmer temperatures may cause insect some species to grow faster, leading to a greater reproductive capacity, which can increase the number of generations produced in a season and the rate of population growth^18^. Global warming will alter the incidence and damage caused by crop pests^19^. Temperature changes also may lead to pest outbreaks^20^. Studies have shown that *T. erytreae* prefers cool and moist climates, because extreme temperatures are detrimental to all stages^21,22^. However, in hot and dry climates or areas that experience high temperatures and steady rain, the pest can grow, but with difficulty^23,24^. It also is believed that *T. erytreae* would be able to survive summer conditions in the Southeastern part of the Iberian Peninsula^21,25,26^. Therefore, understanding the areas at risk of invasion will be helpful for policy formulation, prevention measures, and regulatory plans. Insect pests respond differently to climate change, demonstrating their uniqueness and variances in environmental adaptation^27,28^.

Species distribution modeling (SDM) [also known as ecological niche modeling, habitat modeling, predictive habitat distribution modeling, and range mapping] employs presence records and environmental variables, builds the model using a machine learning algorithm, forecasts species ecological demands, and then projects the analyzed results over time and space to estimate possible potential distribution of the species^29,30^. Species distribution modeling is an important tool in quantitative ecology^31^ and has been used to predict suitable areas for several invasive pests, including *Drosophila suzukii* (Diptera: Drosophilidae)^32^, *Acrosternum* spp. (Hemiptera: Pentatomidae)^33^, and *Helicoverpa armigera* (Hübner, 1808) (Lepidoptera: Noctuidae)^34^.

Species distribution modeling is achieved using either correlative SDMs or mechanistic SDMs. Correlative SDMs use multiple regression techniques to simulate the observed distribution of a species as a function of regionally referenced climatic predictor variables, whereas mechanistic SDMs use physiological data about a species to identify the range of environmental conditions in which the species can survive^35,36^. In correlative SDMs, several models are used, including bioclimatic modeling (BIOCLIM), Boosted Regression Trees (BRT), generalized linear models (GLM), DOMAIN, and maximum entropy (MaxEnt). In contrast, the climate change experiment (CLIMEX) is a semi-mechanistic model that has been used to predict suitable areas of invasive pests^37,38^.

Recent research on *T. erytreae* has focused mainly on its biology^21,22,39–47^, management strategies^48–50^, host range^42^, morphometry^51^, distribution^52^, and citrus greening disease^12,47,53^. Predicting the potential geographical distribution of *T. erytreae* has been limited to a local scale^54,55^, whereas global forecasting of risk areas under current and future climate change scenarios using the CLIMEX model, is generally lacking. Yet, knowledge of the global regions at risk of invasion is required for international collaboration in developing biosecurity and preventive measures to slow down the spread of the pest. In the present study, we combined geospatial and physiological data of *T. erytreae* to predict the global distribution using the CLIMEX model.

## Materials and methods

### Occurrence records of *Trioza erytreae*

We obtained the presence records of *T. erytreae* through field surveys in Kenya, Tanzania, and Uganda, from March 2016 to February 2018. The sampling methods involved the collection of infested shoots, sampling of development stages and adults, deployment of yellow sticky card traps, and sampling of symptomatic shoots. We obtained the GPS coordinates using Garmin eTrex® 32×. Data also were obtained from scientific literature^13,21,22,42–45,47–49,51,52,56–61^), utilizing online databases, such as Web of Science, Science Direct, Google, Google Scholar, PubMed, and MEDLINE. The keywords for global data search on the pest included *Trioza erytreae*, African citrus triozid, African citrus psyllid, ACT, first report, citrus greening vectors, and HLB vector biology, citrus pests, psyllids, and distribution. We also downloaded additional *T. erytreae* data from the Global Biodiversity Information Facility (GBIF, http://www.gbif.org), Centre for Agriculture and Bioscience International (CABI, https://www.cabi.org/), and European and Mediterranean Plant Protection Organization (EPPO, https://gd.eppo.int/). The localities where a village was mentioned, we used Google Earth Pro (version 7.3) (https://earth.google.com) to obtain the latitudes and longitudes. Overall, 301 occurence records were obtained for the modeling. Due to CLIMEX requirements, we cleaned the data by removing duplicates, fuzzy, and neighboring records.

**Figure 1:**
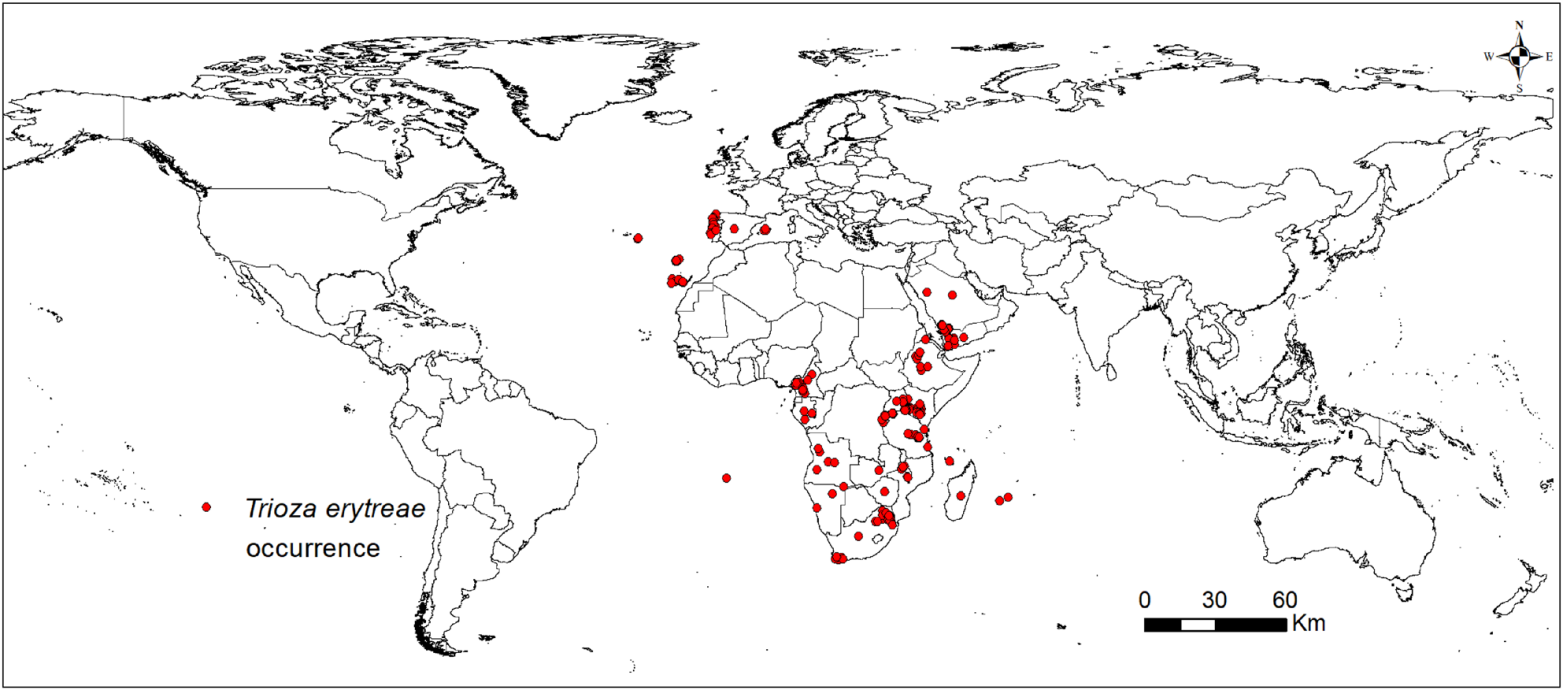
Known global distribution of *Trioza erytreae*. ESRI ArcMap 10.2.2: (https://support.esri.com/en/Products/Desktop/arcgis-desktop/arcmap/10-2-2#downloads).

In addition, the duplicate records from the datasets were removed, leaving just one occurrence per grid cell (5 km). As a result, 283 documented presence records were confirmed and used for the global prediction of *T. erytreae*. The occurrence records used for the model are included in the supplemental material Table S1 and illustrated in Fig. 1.

### CLIMEX model

The potential distribution of a species has been achieved using ecological niche models through semi-mechanistic software such as CLIMEX^62^. CLIMEX is a bioclimatic niche model that forecasts suitable locations based on species traits and climate variables^63,64^. It has been used to predict the potential distribution of various species^65^. CLIMEX is built on the idea that if you know where a species lives, you can estimate its tolerated climatic conditions^64^. As a result, it maximizes the favorable growing season for the species while minimizing the unfavorable growth season^64,66^. This software generates forecasts by combining specific climatic criteria generated from biological data (e.g. heat and moisture requirements) with known species distributions^62,63^. Thus, we created the potential distribution model for *T. erytreae* using CLIMEX. This software provides information on the climate suitability of species, based on stress and growth indices through the Ecoclimatic Index (IE), which is organized on a scale from 0 to 100, where 0 indicates inadequate areas and 100 ideal areas with high suitability for the occurrence of the species^64^. ArcGIS software extracted the CLIMEX outputs and projected the suitable areas for *T. erytreae* in the largest citrus-producing countries, in total production.

### Parameter assembly

The parameters used for the CLIMEX model are presented in Table 1. We adjusted the growth and stress indices in CLIMEX based on scientific research related to *T. erytreae* available in the literature. Based on the best agreement between the observed distribution and the tolerance for climatic variables, the stress indices could be adjusted and classified as satisfactory^67^.

**Table 1:** CLIMEX parameter values are used for *Trioza erytreae.*

Index	Parameter	Mnemonic	Values	References
Temperature	Lower temperature threshold	DV0	10 °C	^68^
	Lower optimum temperature	DV1	15 °C	^22^
	Upper optimum temperature	DV2	32 °C	^63^
	Upper temperature threshold	DV3	38 °C	^63^
Moisture	Lower soil moisture threshold	SM0	0.1	^63^
	Lower optimum soil moisture	SM1	0.3	^63^
	Upper optimum soil moisture	SM2	0.7	Fit to data
	Upper soil moisture threshold	SM3	1.5	Fit to data
Cold stress	Temperature threshold	TTCS	5 °C	Fit to data
	Stress accumulation rate	THCS	− 0.002 week^−1^	Fit to data
Heat stress	Temperature threshold	TTHS	30 °C	^63^
	Stress accumulation rate	THHS	0.002 week^−1^	^63^
Dry stress	Soil moisture threshold	SMDS	0.05	^63^
	Stress accumulation rate	HDS	− 0.005 week^−1^	^63^
Wet stress	Soil moisture threshold	SMWS	2.5	^63,68^
	Stress accumulation rate	HWS	0.002 week^−1^	^63,68^

### Moisture index

*Trioza erytreae* can develop in hot and dry climates like semi-arid areas^68^, thus considering CLIMEX template data of “Semi-arid template,” the Soil moisture indices were defined at a lower soil moisture threshold (SM0) 0.1 and lower optimum soil moisture (SM1) 0.3^69^. The upper optimum soil moisture (SM2) 0.7 and the upper soil moisture threshold (SM3)1.5 were adjusted to agree with the known occurrence of *T. erytreae* and indicate areas with excess soil moisture.

### Temperature index

Eggs and the first instars of *T. erytreae* are highly vulnerable to desiccation, and the lower temperature threshold for the development of the juvenile stages is about 10 °C^68^. Furthermore, temperatures between 15 and 24 °C allowed the development of *T. erytreae* immature stages^22^. Thus, the lower temperature threshold (DV0) was set at 10 °C, the lower optimum temperature (DV1) at 15 °C. The upper optimum temperature (DV2) at 30 °C and the upper temperature threshold (DV3) were set respectively at 38 °C. These indices were adjusted to consider CLIMEX template data of “Semi-arid template” and allow agreement with the high occurrence of *T. erytreae* in semi-arid and arid regions.

### Stress index

#### Cold stress

Temperature significantly affects the development of all the immature stages, with the lowest developmental temperature threshold ranging between 3 and 15 °C^22^. However, the temperature threshold for cold stress (TTCS) was defined as 1 °C, and the accumulation of cold stress (THCS) was set at − 0.002 week^−1^ to fit the pest distribution in the areas of occurrences.

#### Dry stress

Considering the occurrence of *T. erytreae* in many arid and semi-arid regions, we adjusted the dry stress limit (SMDS) to 0.05, and the dry stress accumulation rate (HDS) was set at − 0.005 week^−1^ like the CLIMEX template data of “Semi-arid template”.

#### Wet stress

Although *T. erytreae* populations grow with difficulty, they can also develop in climates characterized by high temperatures and frequent rain, like tropical regions^68^. Therefore, the wet stress parameter (SMWS) was set to 2.5, and the stress accumulation rate (HWS) was set to 0.002 week^−1^ based on CLIMEX Wet Tropical Template.

#### Heat stress

Temperatures of 32 °C or more combined with 30% relative humidity or less kill all stages of the pest^68,69^. Thus, considering the CLIMEX semi-arid template and ensuring the best fit of the model outputs to the *T. erytreae* occurrence, we set the temperature threshold for heat stress (TTHS) to 44 °C and the heat stress accumulation rate (THHS) to 0.002 week^−1^.

### Future climatic scenarios

We used the CliMond 10′ gridded climate data to model CLIMEX since, it provides a good spatial resolution. The CliMond 10′ consists of long-term values of monthly average minimum and maximum temperature (Tmin and Tmax), precipitation (Ptotal), and relative humidity at 09:00 h (RH 09:00) and 15:00 h (RH15:00)^67^. The A1B and A2 SRES scenario and the global climate model (GCM) CSIRO-Mk3.0 (CS) of the Centre for Climate Research, Australia, were used to perform the modelling procedure for *T. erytreae* in the climate changes predicted for 2050 and 2070. The CS climate system model contains a comprehensive representation of the four major components of the climate system (atmosphere, land surface, oceans, and sea-ice), and in its current form is as comprehensive as any of the global coupled models available worldwide^70^.

Our study focuses on two different scenarios, a mitigation scenario (A1B) and a no mitigation scenario (A2). The CliMond database currently does not have data for Representative Concentration Pathway (RCP) scenarios, much less the scenarios are foreseen for the 6th Intergovernmental Panel on Climate Change (IPCC) report that will be released now in 2021. Thus, we used the A1B and A2 SRES latest scenarios present in CliMond. According to Van Vuuren & Carter^71^, the key factor differentiating RCPs from the SRES is their CO_2_ concentration. The A2 SRES assumes an increase in CO_2_ concentrations by the end of the century to 846 ppm. Its best RCP equivalent is the RCP 8.5, which assumes a concentration of 936 ppm. Another difference between these two equivalent scenarios is related to the temperature increase. The predicted temperature increase for the A2 SRES is approximately 6 °C, while for the RCP 8.5 is 7 °C. Therefore, due to its best equivalence to the RCP 8.5, in this study, the A2 SRES scenario was used to model the impact of climate change on *T. erytreae* distribution. Likewise, SRES A1B was used in this study as a scenario more positive, with mitigation actions, equivalence to RCP 2.6.

### Model verification and validation

**Figure 2:**
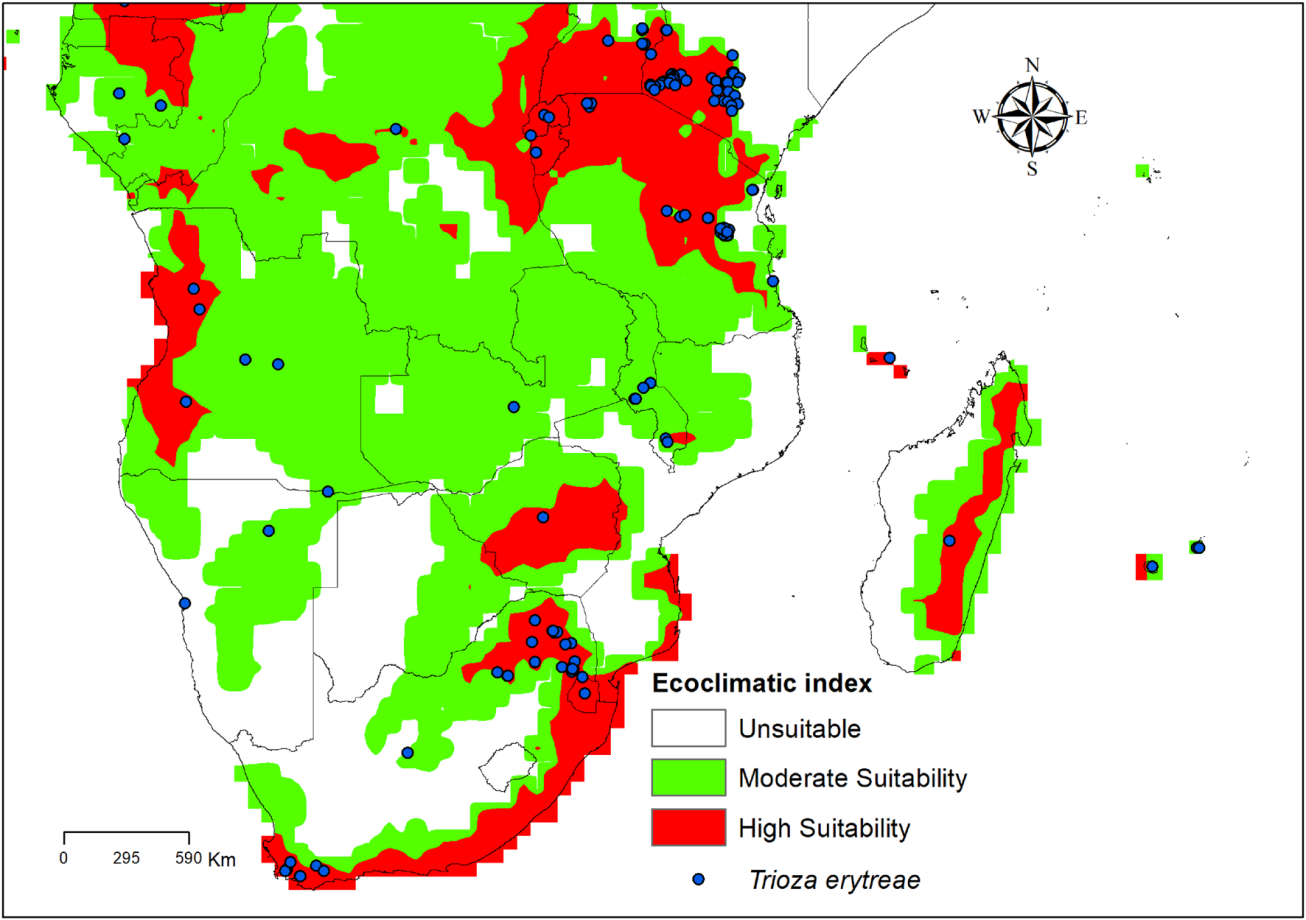
Detail of Known *Trioza erytreae* on the Ecoclimatic index (EI) map from CLIMEX prediction. ESRI ArcMap 10.2.2 (https://support.esri.com/en/Products/Desktop/arcgis-desktop/arcmap/10-2-2#downloads) and CLIMEX 4.0.0 (https://www.hearne.software/Software/CLIMEX-DYMEX/Editions).

The model was validated by comparing the output to known distributions in the South African regions of the Eastern Cape, the first report of *T. erytreae* on citrus^11^ (Fig. 2). These observations were used to evaluate our model’s reliability. Thus, through the Ecoclimatic Index (EI), the suitable areas for *T. erytreae* were classified as unsuitable (EI < 0), moderate suitability (0 < EI < 30), and high suitability (EI > 30)^62^.

## Results

### Current global potential distribution of *Trioza erytreae*

**Figure 3:**
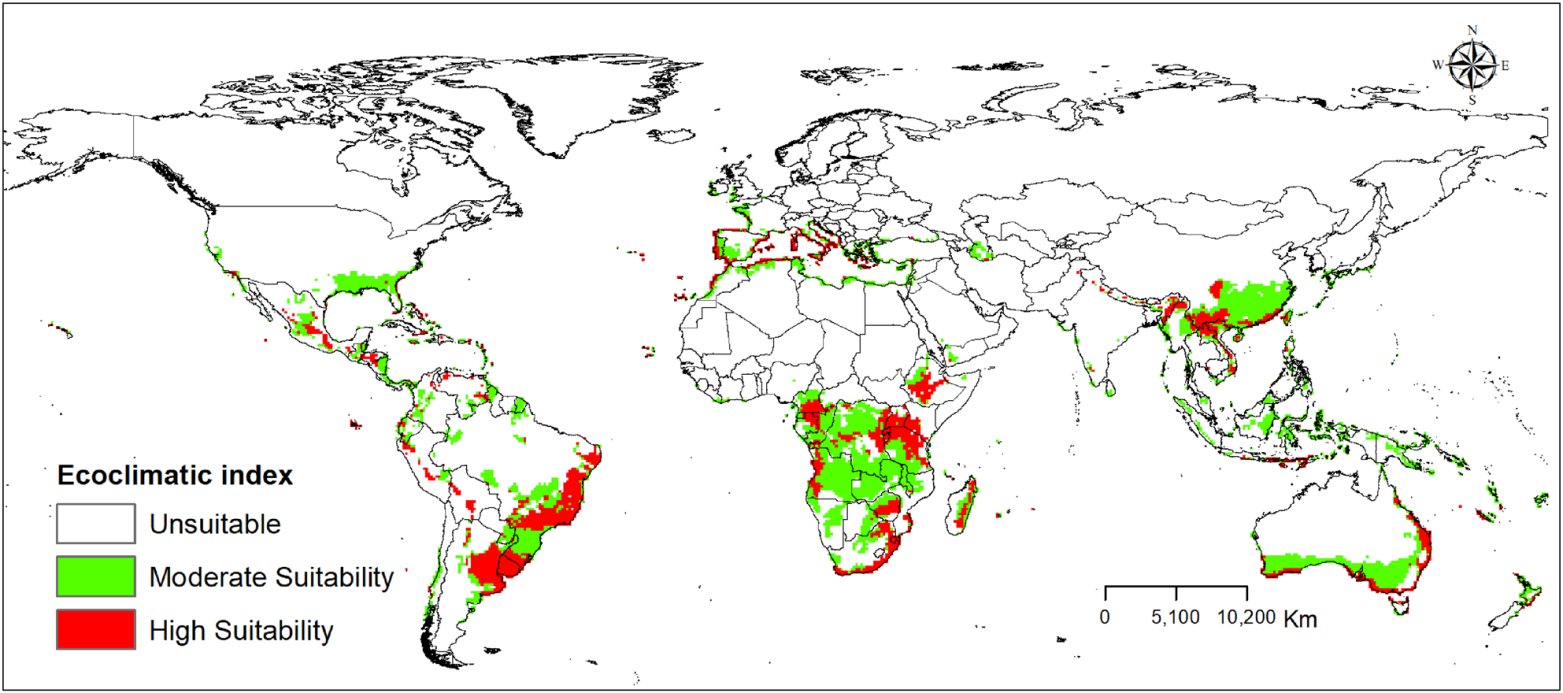
The Ecoclimatic Index (EI) for *Trioza erytreae* is modelled using CLIMEX for the current climate. ESRI ArcMap 10.2.2 (https://support.esri.com/en/Products/Desktop/arcgis-desktop/arcmap/10-2-2#downloads) and Climex 4.0.0 (https://www.hearne.software/Software/CLIMEX-DYMEX/Editions).

The potential geographical distribution of *T. erytreae*, is primarily found in the east and south of Africa; south, and east of Asia; north, west, south, and east of South America, and east and south coasts of Australia. Under the current climate, the Ecoclimatic Index (EI) for *T. erytreae* modelled using the CLIMEX model for the current climate is shown in Fig. 3. The global geographical distribution of *T. erytreae* is consistent with its present-day distributions. However, the model predicts new areas, such as Bostwana, Ghana, Côte D’Ivoire, Nigeria, Liberia, Somalia in Africa; the USA, Mexico, Honduras, Brazil, Bolivia, Paraguay, Uruguay, and Argentina in the Americas; Papua New Guinea and Australia in Oceania; Indonesia, China, India, Thailand, Vietnam, Cambodia, and Myanmar in Asia; Italy in Europe as suitable for the pest.

### Future global potential distribution of *Trioza erytreae*

**Figure 4:**
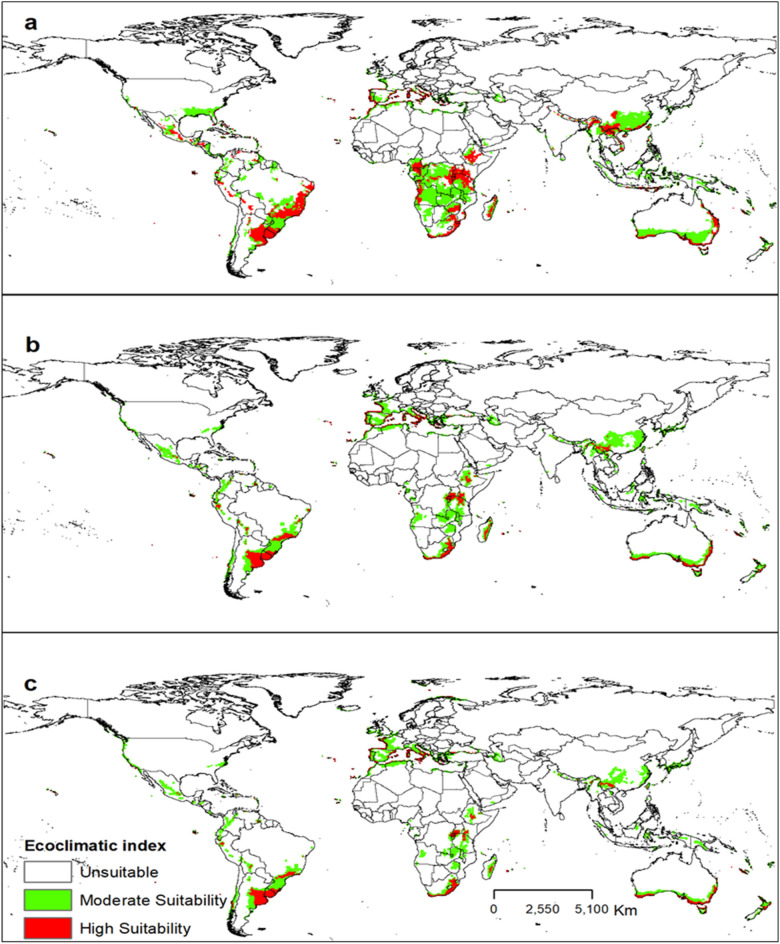
The Ecoclimatic Index (EI) for *Trioza erytreae* using CLIMEX running the SRES A1B scenario for the current time (**a**), 2050 (**b**) and (**c**) 2070. ESRI ArcMap 10.2.2 (https://support.esri.com/en/Products/Desktop/arcgis-desktop/arcmap/10-2-2#downloads) and CLIMEX 4.0.0 (https://www.hearne.software/Software/CLIMEX-DYMEX/Editions).

**Figure 5:**
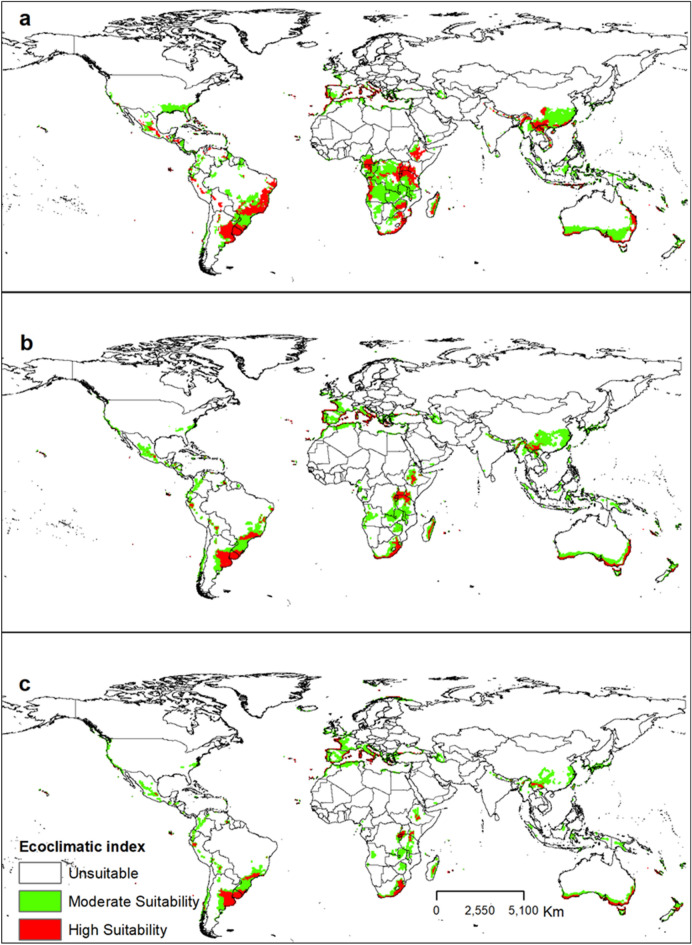
The Ecoclimatic Index (EI) for *Trioza erytreae* modelled using CLIMEX running the SRES A2 scenario for the current time (**a**), 2050 (**b**) and 2070 (**c**). ESRI ArcMap 10.2.2 (https://support.esri.com/en/Products/Desktop/arcgis-desktop/arcmap/10-2-2#downloads) and CLIMEX 4.0.0 (https://www.hearne.software/Software/CLIMEX-DYMEX/Editions).

**Figure 6:**
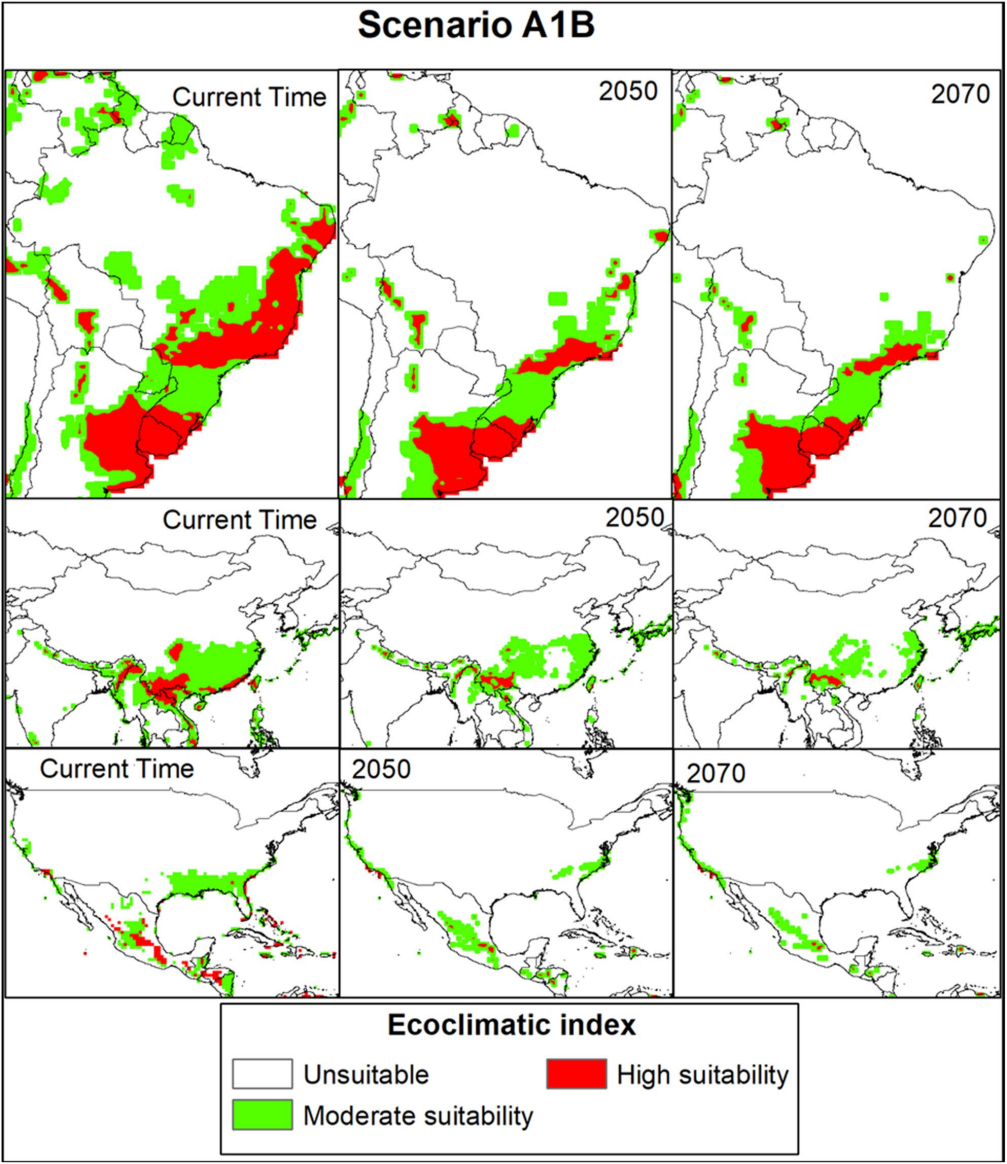
The Ecoclimatic Index (EI) for *Trioza erytreae* in A1B scenario for the current time, 2050 and 2070, respectively, in Brazil, China, and USA (the largest citrus-producing countries). ESRI ArcMap 10.2.2 (https://support.esri.com/en/Products/Desktop/arcgis-desktop/arcmap/10-2-2#downloads) and CLIMEX 4.0.0 (https://www.hearne.software/Software/CLIMEX-DYMEX/Editions).

**Figure 7:**
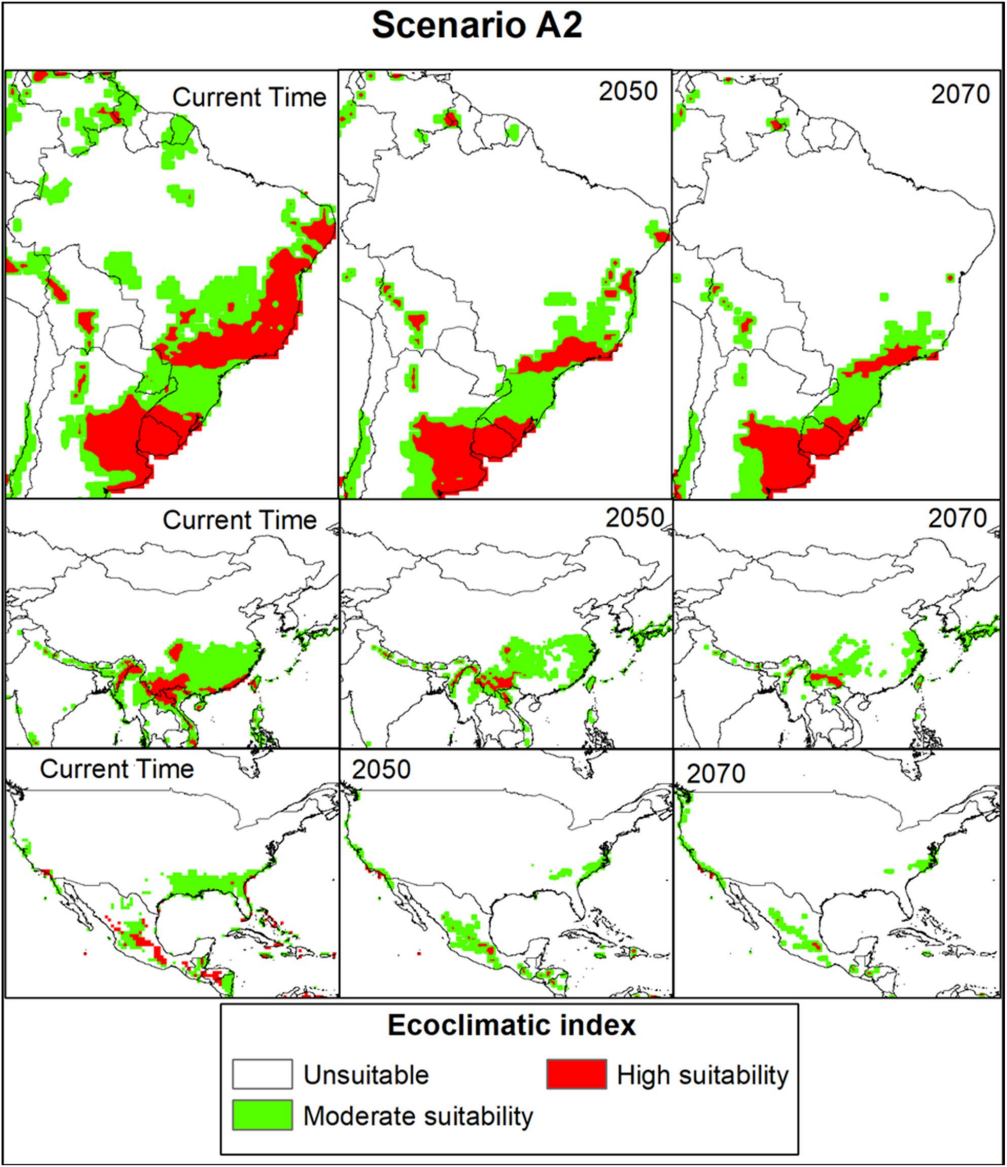
The Ecoclimatic Index (EI) for *Trioza erytreae* in A2 scenario for the current time, 2050 and 2070, respectively, in Brazil, China, and USA (the largest citrus-producing countries). ESRI ArcMap 10.2.2 (https://support.esri.com/en/Products/Desktop/arcgis-desktop/arcmap/10-2-2#downloads) and CLIMEX 4.0.0 (https://www.hearne.software/Software/CLIMEX-DYMEX/Editions).

In the SRES A1B and A2 scenarios, the changes in habitat suitability of the pest from the current time to 2070 are shown in Figs. 4 and 5. The EI for *T. erytreae* in the A1B scenario for the current time to 2070, in the largest citrus-producing countries, in total production (Brazil, China, and the USA), showed a suitable habitat decline for the pest (Fig. 6). The EI for *T. erytreae* in the A2 scenario for the current time to 2070, in the largest citrus-producing countries (Brazil, China, and the USA) (Fig. 7), shows a contraction of suitable areas. The model predicts that highly suitable areas are contered in southeastern parts of Brazil and China. In North America, there will be change of habitat suitability with most parts becoming usuitable for *T. erytreae* in the USA.

## Discussion

In the present study, the physiological data and some of the presence points of ACT were sourced from the scientific literature. This encourages data reuse for future research, allowing future research work to progress more efficiently and effectively^72,73^. Unlike the mechanistic models, the CLIMEX model uses the occurrence records of a species and its physiological stress factors to establish the potential distribution of species. The predictions are consistent with the known global *T. erytreae* distribution. The model predicted suitable areas in regions that were predicted earlier using different machine learning algorithms, such as MaxEnt^54^. Again, the prediction covered suitable areas, including the validation area, in which the data were not used to estimate the geographical distribution of the pest. Overall, of the 283 occurrence points, only one occurrence record was found in areas deemed unsuitable by the model fit, thus most of the occurrence records inside the validation area matched the CLIMEX-estimated suitable areas for *T. erytreae* invasion and spread.

The geographical distribution of invasive species like *T. erytreae* has been evaluated through SDMs, but most studies were conducted locally. For example, Kyalo et al.^54^, combined MaxEnt with remotely-sensed vegetation variables to predict the spatial distribution of *T. erytreae* in Kenya. A study by Aidoo et al.^74^, indicated the potential distribution of *T. erytreae* using bioclimatic variables and elevation datasets in Kenya using MaxEnt. A recent study predicted the expansion of *T. erytreae* in the Iberian Peninsula using a pest risk analysis approach^55^. A more recent study modeled the potentially suitable areas of *T. erytreae* in two European countries, Portugal and Spain using MaxEnt^13^. Finally, Espinosa-Zaragoza et al.^48^, predicted the suitable areas of *T. erytreae* in Mexico based on MaxEnt and only bioclimatic variables. However, none of these studies evaluated the impact of climate change on the global geographical distribution of *T. erytreae*, and under current and future scenarios. Moreover, results from the present study showed similar areas suitable for *T. erytreae* compared to the previous studies^13,48,54,58,74^.

Prevention of invasive alien pests is the surest way of reducing or avoiding their impact on sustainable agriculture production and food security thus prevention of biological invasion is less expensive and more manageable than post-entry management^75^. Therefore, it is essential to understand and assess the impact of climate change on the potential distribution and range shifts of invasive species like *T. erytreae* for a better biosecurity plan. The study's information may be used to develop a proactive, adaptive, and integrated invasive species management approach to curtail and prevent further spread.

In the present study, we used the CLIMEX model to predict habitat suitability for *T. erytreae*, and the suitable areas ranged from unsuitable (EI < 0) to high suitability (EI > 30). The model predicts an expansion of suitable areas outside reported countries in Europe (i.e., Spain and Portugal). These areas were predicted to have moderate (0 < EI < 30) to high suitability (EI > 30) for *T. erytreae*. The new moderately suitable regions identified by the model include Italy, Cyprus, Croatia, Greece, France, and Albania, and these countries produce citrus in Europe; thus the introduction of *T. erytreae* into these zones could threaten their citrus industry. To date, *T. erytreae* is still restricted to Spain and Portugal in Europe^76^. However, the pest can invade and establish in other European countries where citrus is cultivated^55^. This also suggests that there is a need for early detection and eradication measures to avoid the spread of this invasive alien pest across Europe. In Oceania, *T. erytreae* has not been reported, but our study predicts highly suitable areas, particularly in the east and coasts of Australia. If *T. erytreae* were to invade Australia, its establishment would be facilitated by the suitable temperate climate, especially its mild winter temperatures^77,78^. Therefore, continuous strict compliance of the continent biosecurity measures is required at the entry points (i.e., railway stations, harbors, airports, and lorry parks) to intercept host materials. Apart from citrus, alternative host plants belonging to the family Rutaceae, such as the curry tree, *Murraya koenigii* (L.) Spreng. (*Bergera koenigii* L.), and *Clausena anisata* (Willd.) Hook. f. ex Benth.,^74^ should be checked regularly for all stages of *T. erytreae* at the Australian entry points. According to an Australian Plant Biosecurity report^79^, there is a need to pay attention to additional types of invasion pathways, including natural spread, human travelers and their luggage, and infested machinery.

Detection and monitoring of *T. erytreae* using sticky traps can be of great importance to where the pest exists or is at risk of invasion. In Kenya, Aidoo et al.^49^, demonstrated that sticky card traps, especially the yellow ones, effectively detected field populations of *T. erytreae* even at low densities. Other sampling methods, such as visual observation of adults and immatures and collection of symptomatic leaves, could facilitate early detection of the pest, particularly after it has invaded new locations. In most areas, anthropogenic activities have been associated strongly with the spread of *T. erytreae* through movement of infested host plants. Therefore, regular visits to areas where alternate hosts are present may help early detection and monitoring.

We demonstrated that the overall potential habitat suitability for *T. erytreae* will decline in the future under SRES A1B and A2 scenarios compared to the current climate. The A1B and A2 SRES scenarios and the global climate model (GCM) CSIRO-Mk3.0 datasets predict a temperature increase of 2.11 °C and a precipitation loss of 14% by 2100^80^. Previous studies have shown that temperatures above 32 °C coupled with 30% relative humidity degrees are detrimental to *T. erytreae* development^68,69^. As a result, in regions near the equator where the annual mean temperature is around 31 °C, a rise in 2.11 °C may increase *T. erytreae* mortality due to its climatic requirements^80,81^. In contrast, areas with high elevations, such as Kenya, Tanzania in East Africa, will remain suitable because the changing climate may favor the survival and distribution of the pest in those areas. The areas that will see reduction of suitable areas are primarily concentrated in countries such as India and Mexico that are expected to become much warmer. It has been demonstrated that *T. erytreae* prefers cool and moist climatic conditions^24^, so extreme temperature may cause high mortality^22^. On the other hand, the pest has adapted to and settled in a wide range of ecological conditions, including equatorial, arid, and warm temperate climates with varying temperatures and rainfall^21^. In the future, our model predicts more areas of moderate suitability (0 < EI < 30) than high (EI > 30). However, parts of the five major citrus-producing countries in the world (i.e., China, Brazil, Mexico, India, and the USA) will continue to have habitat suitability for the pest until 2070. None of these countries has reported *T. erytreae*^76^. Nevertheless, our study will guide policymakers and plant protection and regulatory services to implement quarantine and preventive measures to avoid future invasion by the pest.

*Trioza erytreae* and the agent it transmits (*C*Laf), are heat-sensitive^68^, and the development of symptomatic leaves varies based on temperature^16^. In addition, detecting the disease in leaves is problematic because ‘*Ca*. L. species’ are of low concentration and unevenly distributed within their host tissues^82,83^. The difficulty in detection may facilitate the spread of *C*Laf in new areas when *T. erytreae* is introduced through pathways, such as citrus fruit^84^, nursery stock (live plants)^85^, budwood^86^, fresh leaves^84^, and grafted trees^87^. Ajene et al.^88^ predicted habitat suitability for *C*Laf in several regions, including Western, Eastern, and sub-Saharan Africa in Africa; South and Central America in the Americas; and the Iberian Peninsula in Europe, Australia in Ocenia, and Cinina in Asia. Similarly, these areas were predicted to be suitable for *T. erytreae*, the disease's primary vector. Because these regions are suitable for both *T. erytreae* and *C*Laf, there is a need to develop proactive measures against the pest and the disease to safeguard the citrus industry^79^. Moreover, in areas where citrus is presently absent, our results can still guide future plans to cultivate the crop, because areas that are currently unsuitable for citrus cultivation may be suitable for its production in future.

It is important to note that there are limiting abiotic and biotic factors, such as natural enemies; entomopathogens, predators, parasitoids; human factors, urban accessibility; and host plant availability; geographical factors; vegetation, land cover, and elevation, that may limit the distribution of the pest, but were not included in the current model. Other factors that were not considered, include competition with native species, altitude, propagule pressure, gene editing, farm-level management strategies, policies, and quarantine measures. All these factors should be considered when interpreting the current study results. Kyalo et al.^54^ found that including remotely-sensed vegetation in a MaxEnt model improved the prediction of suitable areas of *T. erytreae*. Therefore, we recommend that future studies include these factors in the CLIMEX model when predicting the global suitable areas of *T. erytreae*.

As predicted in the current study, the suitable areas in Ghana, Côte D'Ivoire, Liberia, Bostwana, and the Mediterranean coast of North Africa, where citrus is cultivated, require plant protection and regulatory plans. Moreover, these areas need regular inspection or testing for pests, prohibition of portions of the host, a pre-entry or post-entry quarantine system, stated conditions on consignment preparation, specified treatment of the consignment, restrictions on end-use, distribution, and entry periods of the commodity are among the measures that can be implemented^79^. Another way to detect “*Candidatus* Liberibacter spp.” as early as possible is to test the insects^89^. This method has produced results by providing early warning about “*Ca*. L. asiaticus” in several places in the world^89,90^. As a result, a similar approach should be adopted for surveillance of *T. erytreae* by testing all intercepted psyllids.

## Conclusion

The CLIMEX model predictions are consistent with the known historical records of *T. erytreae*, but suitable areas will decrease from the current time to 2070. However, *T. erytreae* suitable areas in some regions will remain highly suitable in the future, implying that major citrus growing countries will continue to be threatened by *T. erytreae* in the future. Therefore, the findings from the present study may help develop preventive and control measures against the pest, especially in regions where *T. erytreae* is absent.

## Supplementary Information

Supplementary Information.

## Data Availability

All data generated or analysed during this study are included in this published article.
